# A Distribution-Free Multi-Factorial Profiler for Harvesting Information from High-Density Screenings

**DOI:** 10.1371/journal.pone.0073275

**Published:** 2013-08-29

**Authors:** George J. Besseris

**Affiliations:** Department of Mechanical Engineering, Advanced Industrial & Manufacturing Systems Program, Technological Educational Institute of Piraeus, Aegaleo, Greece; Universita' del Piemonte Orientale, Italy

## Abstract

Data screening is an indispensable phase in initiating the scientific discovery process. Fractional factorial designs offer quick and economical options for engineering highly-dense structured datasets. Maximum information content is harvested when a selected fractional factorial scheme is driven to saturation while data gathering is suppressed to no replication. A novel multi-factorial profiler is presented that allows screening of saturated-unreplicated designs by decomposing the examined response to its constituent contributions. Partial effects are sliced off systematically from the investigated response to form individual contrasts using simple robust measures. By isolating each time the disturbance attributed solely to a single controlling factor, the Wilcoxon-Mann-Whitney rank stochastics are employed to assign significance. We demonstrate that the proposed profiler possesses its own self-checking mechanism for detecting a potential influence due to fluctuations attributed to the remaining unexplainable error. Main benefits of the method are: 1) easy to grasp, 2) well-explained test-power properties, 3) distribution-free, 4) sparsity-free, 5) calibration-free, 6) simulation-free, 7) easy to implement, and 8) expanded usability to any type and size of multi-factorial screening designs. The method is elucidated with a benchmarked profiling effort for a water filtration process.

## Introduction

Design of Experiments (DOE) furnishes a conceptual interface through which researchers perturb a phenomenon in an attempt to fathom its behavior. DOE also constitutes an indispensable apparatus in any scientific exploration effort that necessitates validation of the gathered information. Being efficient and effective in designing, collecting and explaining experimental observations has been essential in such broad areas spanning from engineering to management while providing a solid basis as the premier research tactic in the core subjects of physical, health and social sciences [1]–[9]. The fundamental framework for enacting a DOE probing as well as the blueprint for data conversion and interpretation have been established by R.A. Fisher [10]. Traditionally, DOE separates in two distinct phases: a) a screening phase and b) a modeling phase [11]. During the process of screening, a group of nominated variables is given a stochastic consideration based on their exhibited potency levels gauged against one or more responses [12]. Only statistically dominant effects are ushered for more detailed manipulation in the model-building phase. From a practical perspective, screening becomes unavoidable when the accumulated costs for executing a series of experiments in a ‘one-factor-at-a-time’ mode appear substantial. Screening might aid to make the cost of acquiring information and thus knowledge more manageable [13]. The economical issues leading to perform screening trials are usually attributed to two well-discerned circumstances. One case relates to the type of experimental set-ups where the sheer forecasted number of the examined factors is projected to a sizable budget. Hence, discovering ways to reduce the amount of work without affecting significantly the information content has been in long pursuit [11]. The second case arises when the cost per trial run is anticipated to be considerable. There are several opportunities in researching operations that may render conducting even a single run an expensive notion. For instance, executing trials that may involve large processing units in production mode may require experimenting with several process inputs. Batch reactors which are used in food production are such large processing units as they are continuous stir-tank reactors which are used in slurry and waste management treatments. In either case, harvesting reliable information from such systems demands tweaking inputs in pragmatic conditions. This in turn entails the consumption of a capacious quantity of materials while simultaneously removing availability from the regularly scheduled operational capacity. This is because a great risk always exists with respect to the fate of the output generated from a completed set of production experiments. At the end of a trial cycle, there is a possibility that the production output would be needed to be scraped and charged along with other production losses. Alternatively, it may happen that the trials incur high costs because of the involvement of pricey novel formulations, in spite of being administered in small amounts. For instance, fuel cell units are loaded with the precious metal platinum. Finally, an obscure reason that makes screening vital in a progressive decision-making process is contingent upon tight project milestone constraints. Collectively, the operational complications mentioned above justify an impetus for more sophisticated data engineering methods that place emphasis on maximizing data mining efficacy in short structured datasets. Inasmuch as screening studies still sustain much demand in several fields [14]–[17], we will focus on proposing a robust profiler that enables information harvesting under imposed extreme sampling conditions. A profiler is to be defined as the stochastic data converter that permits a structured dataset screening. Analyzing datasets designed from high-density factorial schemes maintain an unbridled challenge in science for several decades. High-density screening is to be defined as the profiling performed on a structured dataset where a single datum has been designed-in to contain contributions from multiple effects, in unreplicated mode, while the selected multi-factorial design has been utilized at saturation.

### Fractional factorial screening designs

For a successful screening effort, the choice of the profiler is essential. The profiler may be visualized as the translator that converts data to information. A robust and efficient profiler is one that makes predictions with minimal data, adequate certainty while reliably fending off unknown and unknowable intrusions [18]. Utilizing a robust profiler for screening purposes comprises of two crucial phases: 1) the structured data collection phase, and 2) the data translation phase. In the structured data collection phase, a data engineer formulates a systematic input to be fed to an efficient profiler. In the data translation phase, a selected robust processor sieves out those effects that would constitute significant information. To curtail the data collection effort, researchers have relied for several decades on expertly designed experimental plans. Among the most popular schemes today are the ones derived from high-density data constructs known formally as fractional factorial designs (FFDs) [12]. Intrinsically, FFDs are arrays that have been emanated from the fundamental Hadamard matrix theory. The use of FFD tables is known to reduce dramatically the resources and the execution time required to conduct a complete set of screening trials. While transitioning to higher-density FFDs, it generally holds that the more the investigated factors, the greater the savings incurred.

The process of profiling a group of prospective effects is attempted ordinarily at two predefined endpoints which are determined for each factor individually. The choice of the location of the endpoints may reflect either a tentative or a more legitimate range of interest for the data engineer. The difference between the two is that a tentative range is to be validated posteriori-ly for its suitability, while a legitimate range is usually recommended from known realistic restrictions and expectations. Consequently, the preferred type of FFD arrays which is selected for screening purposes has been predominantly adapted for two-setting (two-level) trials. Popular screening FFDs are those of cubic nature of resolution III or larger (2*_III_^k-p^* or 2*_IV_^k-p^*), and also those of the non-geometric design family of Plackett-Burman [11]. Since the profiler which we propose in the next section does not actually distinguishes between those two basic screening design strategies, for ease of reference, we will denote them collectively from now on as 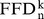. In this generic representation of 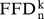, we firstly define the total number of the required recipes or trial combinations to be denoted as *n*. Thus, the number *n* indicates a threshold limit for the size of an engineered dataset such that to permit releasing meaningful information. The superscript *k* in the 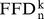 symbolism denotes the maximum number of examined influences that may be handled by a given plan when each effect is to be tweaked individually at two specific operating endpoints.

### A brief account of the properties of high-density 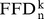 in profiling

**Figure 1:**
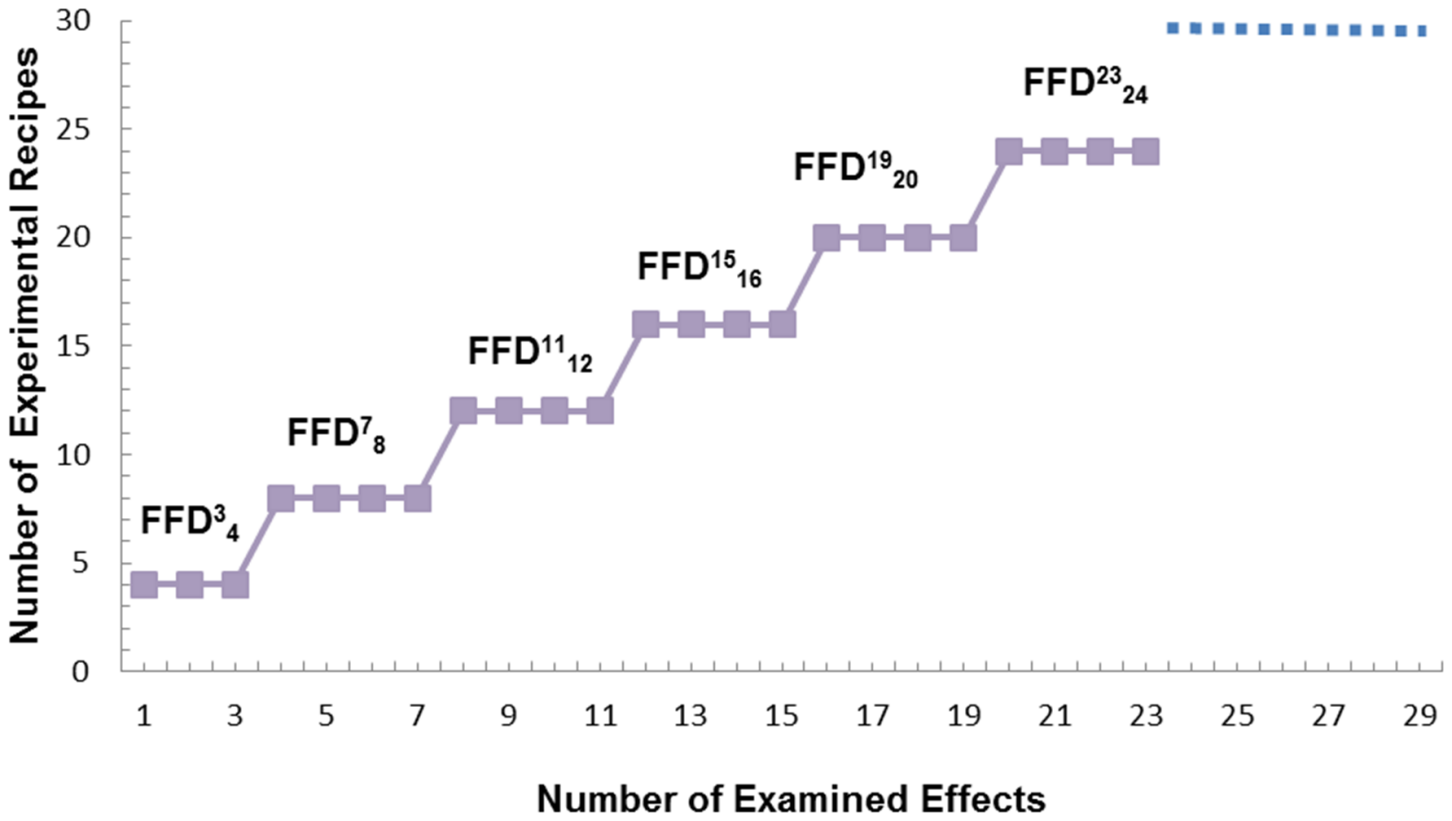
The quantized plateau for increasing trial-run capacity in 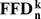 schemes: Graphing the experimental recipe count versus the number of examined effects.

A few inherent properties of the 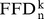 arrays that reveal the idiosyncrasies of such schemes are now briefly addressed. Firstly, the uptake of controlling factors in a given 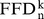 plan is quantized such that the required recipes are populated in multiples of four [12]. The quantization rule may be expressed as *k* = *n*−1 with *n* = 4· *i* (*i* = 1, 2, 3…). This immediately implies that for a given 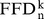, accommodation of a group of any *m* investigated effects in the experimental scheme is enabled subject to the restriction: *k*−3 ≤*m*≤*k*. This is further interpreted to mean that the number of trials reaches a plateau until the 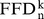 array progressively fills up with assigned factors up to the maximum capacity of *k* variables (Fig. 1). If *m* = *k* then the array is defined to have achieved a ‘saturated’ state [11]. While driven to saturation, the experimental design is primed now to allow extracting maximum information for the effort expanded. This is because a saturated 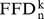 engages all its available array columns by assigning them in full to the respective investigated factors. As a result, experts scrutinizing different strategies to engineer optimal experimental plans have recognized long ago that ‘saturation’ may be a desirable condition for maximizing the performance of information harvesting with minimum cost [19]–[23]. Likewise, it was discovered that additional gains in cost and time could be anticipated by potentially eliminating data replication at all [24]–[30]. Unreplicated trials simply constitute a single execution of a specified set of recipes as dictated by a given 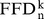 plan. Clearly, the conditions of conducting either unreplicated or saturated trials are distinctly different. However, the specialized methods which have been devised for handling either of the two aforementioned situations have been misconstrued to be employed interchangeably [31]–[32]. We clarify at this point that it is trivial to manipulate a saturated 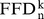 dataset which has been replicated using mainstream statistical tools as much as it is possible to treat straightforwardly an unreplicated dataset that is not saturated. For either case, the researcher has several options to treat the experimental data starting with the standard multi-factorial comparison methods, such as the Analysis of Variance (ANOVA) along with the multi-parameter regression-type solvers abiding to the classical General Linear Model (GLM).

It is only when analyzing 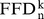 datasets that have synchronously been subjected to saturation and unreplication that subsequent data manipulation and decision making become an intriguing and perplexing affair [33]–[34]. A singularity emerges during the conversion stage of a saturated-unreplicated 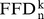 dataset whenever treated with regular multifactorial solvers. Such singularity is formed because of an inherent incompatibility of the standard data processors (ANOVA or GLM) to cross-match their required degrees of freedom (DFs) with those DFs provided by the corresponding unreplicated-saturated 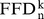
dataset. Techniques based on the ANOVA or GLM framework are tuned to release statistical significance evaluations only when the unexplainable error is estimable. However, the unexplainable error is a quotient which in order to be computable, its divisor, i.e. the remaining DFs not absorbed by the investigated effects, should be a non-zero quantity. If the total number of remaining DFs adds up to zero, then, the unexplainable error obviously manifests a singularity forcing the data processor to crash. Such complication always occurs in analyzing saturated-unreplicated 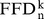 plans, because all available DFs are exclusively allotted in order to form the variances of the investigated effects. In other words, there are in total *n*−1 DFs to be distributed over to the *k*-effects (*k* DFs). But for saturated-unreplicated 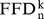 schemes, the relationship among DFs is that *k = n*−1. Thus, there are no remaining DFs to estimate the unexplainable error. This serious drawback that appears in the data analysis step for treating saturated-unreplicated 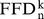 became the subject for a benchmark study where twenty-four novel and hybrid methods were compared for their efficacy producing no particular frontrunner [32]. Since then, the interest for finding an all-around compatible solver has remained unabated. A more recent survey about the progress in discovering new techniques may be assembled through several references [34]–[36]. By convention, from now on, when we refer to high-density screening designs we intend to mean saturated-unreplicated 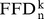.

### Motivating the deployment of high-density 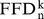 in profiling

#### Embracing the exploitability of 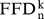


**Figure 2:**
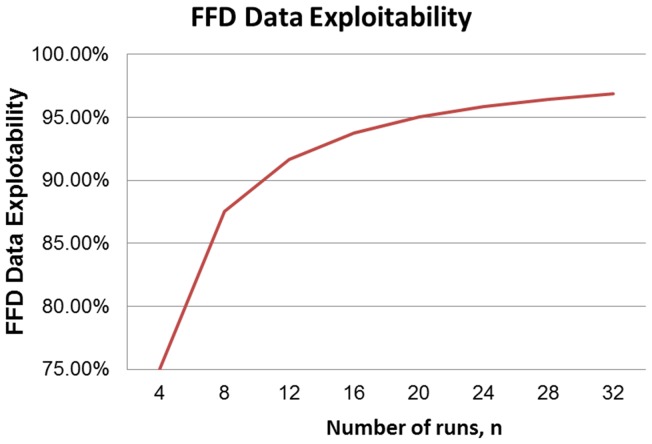
Exploitability performance in terms of increasing number of runs.

**Figure 3:**
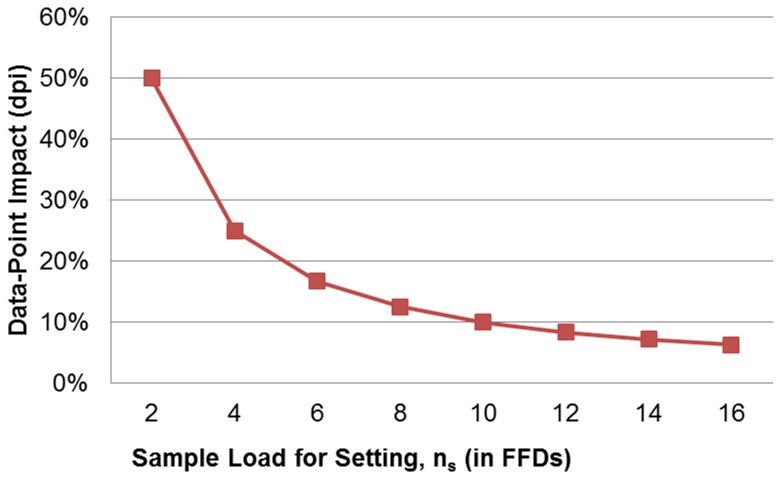
Data-point impact versus sample load for factor-settings in FFDs.

Expenditures allocated for generation of new knowledge are dramatically suppressed when profiling effects through 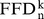 screening. This is better elucidated if we introduce one practical, yet meaningful, indicator. Such indicator should reflect the utilization of a dataset for testing a number of effects in a given saturated-unreplicated scheme. We define the exploitability (X^a^) metric based on the number of experimental combinations to be executed, *n*, and the number of considered effects, *k*, to be: X^a^ = *k*/*n*. The exploitability is expressed in%. Obviously, the higher the exploitability, the more efficient the data engineering phase. In other words, a saturated-replicated 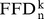 scheme with a higher exploitability rate makes more efficient use of the generated data in the process of extracting information. The best way to comprehend the importance of exploitability is to view the performance of some of the most popular schemes. For illustrational purposes, in Table 1, we list a performance comparison for the exploitability rates of the first eight consecutive 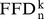 family members. Additionally, in the same table, we tabulate the corresponding exploitability performance for the respective full-factorial counter-scenario while still considering the same two-level, *k*-effect screening. It becomes obvious now that as the size of the 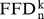
array increases, exploitability grows, thus reflecting a more efficient use of the available data in gauging even greater number of effects. On the contrary, it is striking that settling for the naive full-factorial design option leads to prohibitive low levels of exploitability. The explosive demand for data which is deeply ingrained in full-factorial systems intensifies as the number of considered influences increases. We now define the data volume reduction quotient, Q, which represents the factor by which the data volume in the data engineering phase is curtailed by selecting a proper 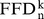 plan rather than deploying an ordinary full-factorial scheme. The astonishing data requirement reduction elicited by utilizing, for example, the 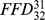 data-planner over its corresponding full-factorial may not be overstated (Table 1). Another important trend that arises in Table 1 regarding the behavior of 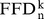 is that as the number of experiments, *n*, increases, the data volume committed for a single effect also increases. Therefore, there is an inherent motivation for discovering more efficient and effective processors for converting data generated from smaller-sized FFDs. Finally, we observe that as the size of the FFD plan increases, exploitability tapers off. This behavior is better discerned from Figure 2, where we graph the data from Table 1, placing in the abscissa axis the size of the FFD, *n*, and in the ordinate axis the respective exploitability. Now, the leveling off of the exploitability for larger sized arrays becomes more evident while the slope dwindles monotonically. However, scientifically, it is of great interest to develop high-fidelity profilers at low exploitability and low sampling limits. From Table 1 and Figure 2, there is indeed a great motivation to work the range for the first four 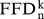 family members, covering the size range of 4 to 16 trials (3 to 15 parameters). For instance, the 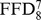 plan is particularly attractive because: 1) it is sizable enough to be utilized in many applications in the general scientific and engineering literature, 2) it possesses a reasonable exploitability while it is situated in the fastest transitioning zone (Figure 2) and 3) it is admittedly a low sampling scheme demanding only four data points per examined factor setting. It is for those reasons that we selected to demonstrate the applicability of a new robust data converter, later in this article, by adopting a case study that engages a 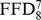 plan.

**Table 1:** Exploitability for several common 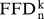 screening designs.

Screening Design	Maximum number of factors	Total number of FFD runs	Total number of full-factorial runs	Data Volume Reduction Quotient	Full-factorial Exploitability	FFD Exploitability
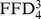	3	4	8	2	37.500000%	75.00%
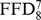	7	8	128	16	5.468750%	87.50%
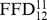	11	12	2048	171	0.537109%	91.67%
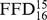	15	16	32768	2048	0.045776%	93.75%
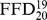	19	20	524288	26214	0.003624%	95.00%
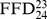	23	24	8388608	349525	0.000274%	95.83%
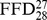	27	28	134217728	4793490	0.000020%	96.43%
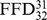	31	32	2147483648	67108864	0.000001%	96.88%

#### The data-point impact in 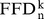


A useful measure in dense-data engineering could be proposed through the data-point impact index (dpi). Dpi could be taken to define the weight of any given single data point in the collected sample to a forthcoming analysis effort. It comes naturally to define such a metric for 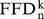 samplers by virtue of knowing in advance that a single data-point is the outgrowth of as many as *k* concurrent influences captured in a single measurement.

In the FFD framework, the aspect of sample size mirrors a predefined demand for balancing the requisite data volume among the studied effects. Sample points should be equally distributed to their respective factor settings. A measure that may describe the relative data requirement for a given 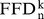 sampler should be the sample load for a factor setting, *n_s_*. For the 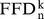 family of two-level sampling plans, it is straightforward that it holds that *n_s_* = *n*/2. We now define dpi to be: dpi = 1/*n_s_*. The dpi indicator will be expressed in a percentage scale. It is understood now that the higher the dpi value, the more each data point is contributing in shaping information for a particular 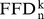
scheme. In Figure 3, we plot the trend of dpi against the sample load for a setting by portraying the behavior of the first eight indicative 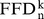 plans of Table 1. It is transparent from Figure 3, that it is the samplers attached to lower *n_s_* values that should attract the most attention because they shoulder the higher dpi ratings. The change of slope in the same graph is greatest for the 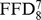
scheme implying that exploring data conversion for such high dpi schemes should be intriguing.

#### In search for an all-purpose, robust profiler for 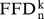
samplers

Computing location and dispersion estimators offer a way to measure sample central tendency and variation in a structured dataset. In the in-depth research of Hamada and Balakrishnan [32], one of the insightful recommendations that were raised foresaw that the impetus of future developments in analyzing unreplicated fractional factorial schemes would be spearheaded by non-parametrics. Order statistics are known to offer robust data reduction capabilities even when the underlying data distributions persist to remaining hidden during the conversion process. Therefore, enforcing distribution-free analytics could aid in by-passing unresolved data structure issues manifested exclusively in unreplicated-saturated multi-factorial sampling plans. One such major issue emerges by the indeterminate nature of the experimental error in ANOVA or GLM type of analyzers. We mentioned previously that the absence of a residual error stonewalls the solver effectiveness. Ergo, there were several attempts since the Hamada and Balakrishnan [32] report to adapt various non-parametric solvers in explaining densely-packed factorial data [33]–[34],[37]–[38]. Nonparametric methods are routinely used in management to model behavior when data is scarce [39]. Up until now, non-parametric combinatorial algorithms are: 1) intensely customized and 2) geared towards some limited and preselected 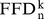 family members. A generalized strategy to handle every type of 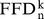 s has not been attained to this day. The instilled agility of order statistics has not been taken advantage in full neither their lean reporting capability. There are several desirable requirements for novel profilers intended to harvesting information from high-density samplers. Effective profilers should include the following features: 1) robustness, 2) versatility, 3) adaptability, 4) ease to deployment, 5) calibration-free, and 6) simulation-free. Much sought-after feature for competitive solvers will further embody a sparsity-free framework. Sparsity requires sacrificing some effects to play the inescapable role of weak effects. Rounding up weak effects is a subjective action to assist leveraging out significant influences. Thereby, weak effects are masked contributions primed to play the role of the elusive residual error in ANOVA (or GLM)-type treatments [34]. Opting out the sparsity condition from a statistical engine assures the objectiveness in the filtering process. Robust information is gleaned only when the solution is not challenged by the extent of the sparsity which should be present in the model. Furthermore, high-density data converters should possess an acceptable performance with regards to Type I and II statistical errors which should be nevertheless easily verifiable and accessible even to non-experts.

## Materials and Methods

### A new non-parametric methodology for treating high-density 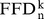


We consider a generalized saturated-unreplicated screening design, 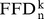, for testing a response *Y*. Such design assigns all of its available *k* columns to as many as *k* controlling factors - including possibly interactions - for programming the required set of *n* unique trial recipes. Therefore, the unreplicated dataset for the *n* conducted trials is composed of a ‘one-time’ group of observations which are denoted as 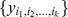. Each *i_j_* (*j* = 1, 2,…, *k*) in the notation identifies with a corresponding investigated influence. It holds for all *i_j_*_s_ that there are only two admissible states for any given factor. Those are the search states that will reflect the behavior of the investigated response at the two predefined endpoint settings. For any two-level screening design, the two tested endpoints are traditionally symbolized in literature in a simply coded form which is marked respectively as: ‘−’ and ‘+’ [11]. We present now an effects model that accounts also for the presence of a generic unexplainable contribution. The unexplainable contribution,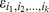, consists of a random error plus any other spontaneous unknowable intrusions. Assuming that the number of the tested factors (and/or interactions) is *k*, then, the effects model may be expressed as:
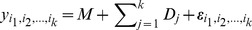
(1)


The overall (grand) median, *M*, in equation 1, is defined for all *n* collected observations as:
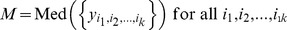
(2)


Before we elaborate on the summation term regarding the indexed quantity *D_j_* in equation 1, it is prerequisite to define previously the partial (setting) median values 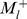 and 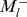
with 1≤*l*≤*k*. The partial median values represent a median estimation of the values for each group of observations that have been treated at the same factor setting *i_l_* (1≤*l*≤*k*). To simplify the notation in our statistical model, we define the general partial median, 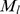, with the following property:
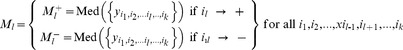
(3)


The difference between 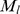
and *M* isolates the *i_l_* ^th^ partial effect due to the *l* ^th^ factor with respect to the grand median. This difference will be denoted as *D_l_* (eq. 4) and it is the quantity that enters the summation operation in equation 1.
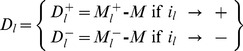
(4)


As soon as the model in equation 1 has been fitted, we may separate the contribution of each effect by forming a new pseudo response, 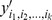. The pseudo response is simply reconstructed in terms of the grand median, *M* (eq. 1), the partial effect, *D_l_*, and the corresponding error contribution, 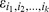
for all *i_l_*. Hence, the new pseudo-response which is configured from that last prescription has the following stochastic structure:
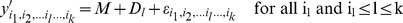
(5)


The next step is to simply rank-order 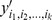
in the usual manner such that the pseudo-response is transformed to the new rank response,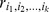:
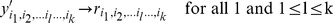
(6)


The process of rank ordering may be initiated to any arbitrary direction. For ease of reference, we establish a convention by commencing enumerating ranks to pseudo-response entries such that to coincide with those observations which are identified with higher performance. If a characteristic ought to be conditioned to perform at its maximum response, then we start assigning the lowest rank (1) to the observation with the maximum magnitude completing the ranking by assigning the largest rank value (*n*) to the smallest observation. On the reverse, if it is desirable to minimize the magnitude of a characteristic then the lowest rank is allotted to the lowest observation and so forth. Finally, there is a third case where it may be sought a characteristic value that converges as close as possible to a specified target value. Then, the absolute difference of the response from the target value is treated according to the second case we just mentioned. After the ranking operation has been completed, the rank-sum method for a two-sample unpaired comparison of Wilcoxon [40] and, Mann and Whitney [41] (WMW) may be deployed to stochastically determine if the level-splitting caused for any given *l*-effect is indeed significant. The rank-sum estimator, *T_l_*, will be suitably adapted for gauging the performance of the pseudo-response due to the *l* ^th^-factor at its two initialized settings. Therefore, *T_l_* will be defined by convention as the minimum of the two rank-sum quantities associated with the two estimates at the two corresponding operating endpoints, 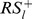 and 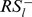: 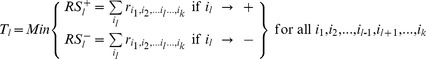
(7)


The null hypothesis (*H_o_*) assumes that all effects are ignorable for all *T_l_* (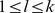) estimations. The well-known WMW reference distributions will provide the cut-off points for statistical significance whilst profiling each effect separately. The reformulation of the original response to *l*-individual pseudo-responses permits the extension of the versatility of the (single-factorial) WMW statistics to the much broader class of multi-factorial 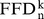 samplers. In case there is a manifestation of ties in the treated pseudo-response data, then we resolve this issue by resorting to the Hemelrijk method [42]. To quantify the significance of fluctuations of the residual error due to different individual contrasts, a new type of response is created which is appropriately declared as the residual response, 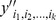 for 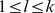 (eq. 8). The residual response is created by simply retaining the grand median of the original data and the error contribution for each trial run:
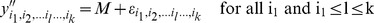
(8)


The same tactics are followed here as it was proposed previously for the analysis of the pseudo-response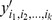. Hence, a corresponding rank ordering will yield the newly transformed rank-residual-response,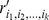:
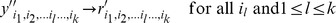
(9)


The rank-sum estimator for testing the residuals dependency on the *l*-effect,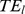 is simply the minimum of the split rank-sums at their respective settings, 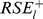 and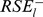. The formalism for treating residual rank-sums is:
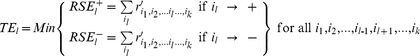
(10)


Now, we may probe the residual response behavior for prominent effects according to the same WMW reference distributions. The null hypothesis (*H_o_*) declares that all 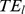
(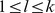) are expected to be statistically insignificant. As a final note, we clarify that we maintained the grand median term as a matter of style in all reformed response configurations which were introduced in this section in order to sustain a homogeneous (positive) sign in all restructured data entries.

### Profiling a complex water filtration process

Water is a critical resource for survival on this planet [43]. Therefore, sustaining water purity while managing depletion prevention ranks among the top world priorities in business today [44]. Water filtration is a common as well as a complex process to deal with. Filtration is also selected as a subject to demonstrate robust profiler techniques because it is a process easy to comprehend intuitively as it is encountered often in multifarious industrial environments and other scientific laboratories where new information is chronically produced. The mechanisms that control filtration are known to be complex in the sense that they may also be characterized in terms of fractal dimensions in addition to other random intrusions [45]. The published industrial filtration screening in Box et al. [11] will be discussed since it has been scrutinized in the recent past in at least three studies; the other two being by Miller [46] and Besseris [34]. The selected case study aids in exposing the flexibility that it is expected in supporting decision making with robust inferential tools. In this direction, the study showcases how to transform a highly dense dataset created by a group of categorical inputs, adjusted from nominal scaling, even though the response is of a continuous numerical data type. In brief, seven factors are tested using a 2_III_^7-4^ screening design (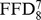). Thus, the compressed dataset has been generated by reducing by a fraction of 1/16 the expected full factorial requirement of 128 trials. Each controlling factor setting contributes only four data points to the conversion process for gaining pre-structured information. The combination of engineering a low sampling condition into a highly-compressed data-collection scheme should consequently pose towering demands on the data processor effectiveness. The profiling of the fractionated filtration process is compelling because we identify with this situation both conditions that intensify the extent of any potential discovery. We remind that the engineered dataset has been programmed to collect concurrently response readings in the unreplicated and saturated form. The original data are reproduced in Table 2 in terms of the respective controlling factors and their corresponding coded settings. The pertinent variables are: the water supply source (WS), the raw material origin (RM), the temperature setting (T), the recycle choice (RE), the filter cloth type (FC) and the holdup time (HT). The physical meaning of the respective inputs in terms of the standard symbols ‘−’ and ‘+’ is easily accessed in Box et al. [11]. An inputs list has been recreated in Table S1 for readers' convenience. The examined response is the filtration time (FT) recorded in minutes. A desirable FT quantity should result from minimization of the response. The original data will be manipulated with mainstream techniques in order to assist in demonstrating the benefits of the proposed approach. Therefore, in the Discussion section we make comparisons of our method with standard techniques such as 1) the ANOVA method, 2) the main effects plot, 3) half-normal plot and 4) Pareto chart supplemented with Lenth test critical values. All statistical analysis output regarding those four methods along with their accompanying graphical depiction of the performance of the contrasted effects have been generated though the use of the software package MINITAB 16.1.

**Table 2:** The 2_III_^7-4^ screening design for filtration rate data [11].

run #	WS	RM	T	RE	CS	FC	HT	FT
1	−	−	−	+	+	+	−	68.4
2	+	−	−	−	−	+	+	77.7
3	−	+	−	−	+	−	+	66.4
4	+	+	−	+	−	−	−	81
5	−	−	+	+	−	−	+	78.6
6	+	−	+	−	+	−	−	41.2
7	−	+	+	−	−	+	−	68.7
8	+	+	+	+	+	+	+	38.7

## Results

In Table 3, the partial median values per factor setting (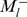,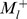) have been computed from the original dataset (Table 2) and tabulated for all implicated factors individually. The corresponding partial effects due to the two settings (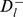,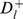) are also displayed on the same table where the differences were calculated with respect to the grand median value of 68.55 minutes. In Table 4, we provide the reconfigured datasets for the respective pseudo-responses (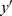) and the accompanying residual response (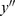). The corresponding rank transformations resulting to the pseudo-responses (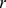) and to the residual response (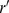) are highlighted in Table 4 in red-colored font.

**Table 3:** Order stochastic estimations for filtration data from Table 2.

*l*-effect	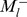	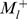	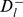	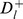	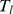	*p_T_*	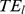	*p_TE_*
WS	68.55	59.5	0	−9.05	10	0.029	14	0.345
RM	73.05	67.55	4.5	−1	17	0.886	14	0.345
T	73.05	54.95	4.5	−13.6	10	0.029	16	0.686
RE	67.55	73.5	−1	4.95	13	0.200	14	0.345
CS	78.15	53.8	9.6	−14.75	10	0.029	14	0.345
FC	72.5	68.55	3.95	0	12	0.114	18	1.000
HT	68.55	72.05	0	3.5	17	0.886	14	0.345

**Table 4:** Reconfigured data from Table 2 in terms of pseudo-responses (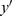), the residual response (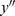), and their associated rank values (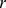,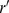).

	PSEUDO-RESPONSE 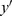								RANKED PSEUDO-RESPONSE, 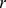							
run #	CS	T	WS	RE	RM	FC	HT	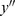	CS	T	WS	RE	RM	FC	HT	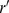
1	54.45	73.7	69.2	74.15	92.25	69.2	69.2	**69.2**	2	7	6	8	8	3	3.5	**5**
2	75.25	70.15	56.6	64.65	64.65	65.65	69.15	**65.65**	5	5	1	1	3	1	2	**1**
3	56.45	75.7	71.2	70.2	64.7	75.15	74.7	**71.2**	4	8	7	3	4	8	7.5	**7**
4	77.65	72.55	59	73	85.6	72	68.05	**68.05**	7	6	2	5	7	5	1	**3**
5	75.3	52.1	65.7	70.65	76.15	69.65	69.2	**65.7**	6	1	5	4	6	4	3.5	**2**
6	56.4	57.55	62.1	70.15	57.55	75.1	71.15	**71.15**	3	3	4	2	2	7	5	**6**
7	84.3	61.1	74.7	73.7	55.6	74.7	74.7	**74.7**	8	4	8	7	1	6	7.5	**8**
8	53.9	55.05	59.6	73.6	73.6	68.65	72.15	**68.65**	1	2	3	6	5	2	6	**4**

Now, we provide an illustration of how to sequence ranks based, for instance, on the CS pseudo-response (Table 4). We recall first that the filtration rate is a characteristic that needs to be minimized. Assigning ranks commences by allocating the rank of 1 to the smallest (8^th^ run) entry value (53.9 minutes), reading off from the CS column organized properly in the group of pseudo-responses,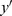, in the left part of the Table 4. Incrementing the rank assignment terminates with the allocation of the least (7^th^ run) desirable entry value (84.3 minutes) which will be awarded a rank of value equal to 8. Thus, the rank column that reflects the isolated, and now ordered, CS-dependent-only FT-response is tabulated on the right-hand part of Table 4 under the proper heading (CS) in the grouped data columns in the *r*-responses section. After Table 4 has been completed by assigning ranks to all pseudo-responses including to the residual vector (*r*″), the rank-sums (*T_l_*) are computed for each pseudo-response separately thus furnishing the potency levels of each effect (Table 2). For example, to form the rank-sum *T_T_* which is attributed to the T-effect, we need to form first the two partial rank-sums, 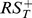 and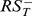, respectively. By inspecting the original array (Table 2), it is prescribed for the T-factor that the first four entries represent the factor-level (−) while the remaining four entries are attributable to level (+). From Table 4, we proceed to estimate the magnitudes of 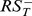 and 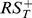 which equal 26 ( = 7+5+8+6) and 10 ( = 1+3+4+2), respectively. Hence, the rank-sum *T_T_* equals to the minimum of the two rank settings, i.e. to a value of 10. Similarly, all the rank-sum values are calculated for the remaining six effects and they are listed in Table 3 along with the performance of the residuals for *TE_l_*. Table 3 is completed by tabulating the respective p-values for the rank-sums which have been retrieved from the WMW reference distributions. They are separately listed for each of the seven considered effects (*p_T_*) and their corresponding residuals (*p_TE_*). It stands out that the residuals do not interfere asymmetrically to any of the seven considered effects since their *p_TE_*–values were well above the cut-off significance level of 0.1. This simply means that any underlying ‘unknown and unknowable’ contributions are equally distributed (statistically) across all entries. Therefore, all ‘unpaired-wise’ comparisons within each effect may be rendered meaningful. Interpreting our profiling manifestations, the three factors that perform stochastically superior to the rest of the group are: WS, T and CS, when contrasted at a typical individual error rate of 0.05. Surprisingly, all three effects post identical *p_T_*-rates of 0.029. The experimentwise error rate [47] for this case study is 0.10 (in two significant figures) which is deemed exceptional for such a high-density trial scheduling. From Table 3, the settings that seem to favor the minimization of the filtration time are WS^+^, T^+^, and CS^+^ which mirror to median response values of 59.5, 54.95 and 53.8 minutes, respectively. In turn, it is decided that the preferred filtration system set-up should use well water supply in a high temperature environment while dispensing slowly amounts of caustic soda.

## Discussion

Analyzing saturated and unreplicated fractional factorials has been in long pursuit for several decades without being ever convincingly resolved. The tremendous importance of explaining ‘high-density’ experimental protocols is tangent to any aspect in science. Past studies have revealed that there was no convergence on a single unified approach in spite of an overwhelming number of individual attacks of treating either the saturated or the unreplicated condition [12], [32]. In this article, an all-purpose fractional factorial profiler has been proposed for deciphering an efficient saturated-unreplicated high-density screening. Our suggested profiler is a robust data converter that sieves through harvested information to stamp significant effects. Inasmuch as our approach may be considered a breakthrough in bridging the gap between factorial-data compatibility and distribution-free analytics, the overall concept remains simple, practical and easily verifiable. Our approach literally manages to ‘slice-and-dice’ structured data generated from high-density experimental plans. To achieve a terminal result, our method ignores an upfront stipulation for designating the size of the residual error. Conventional multi-factorial data-processors - ANOVA or GLM - have been devised to deliver a statistical rate of occurrence only when the underlying unknown and unknowable phenomena have been rounded-up and accrued into a single variance. It is the variability of the unknown and unknowable that forms the ‘measure stick’ on which the variances of the examined effects will be gauged against in ordinary ANOVA or GLM treatments. Unfortunately, the highly-efficient saturated-unreplicated factorial designs - when implemented for engineering dense datasets - upset the very essence of the ANOVA (or GLM) converter by dismantling that ‘measure stick’. Ergo, ANOVA (or GLM) is disabled to streamline an error-variance evaluation to subsequent stochastic contrasting (through the F-test). As a result, ANOVA succumbs by coercing the processor to impasse. On the contrary, our profiler proceeds to metamorphose dense data to information without the need for weighing each of the examined effects against an explicit ‘measure scale’ for error. It is remarkable that our profiler while it maintains its pure nonparametric character in all analysis steps, it does not require any customized combinatorics routines or novel simulations. Thus, our data miner is not selectively applicable to only some types of factorials [34], [46]. It is emphasized that the great discovery here is that it is not necessitated to devise a new stochastic framework with assorted novel reference distributions. Our selected data processor is solidified upon the well-tested WMW robust stochastics. WMW theory has been studied, implemented and explained for more than half a century now to a diverse range of applications in science and engineering [34]. Consequently, performance issues as well as test power concerns about our method are now inherently identifiable with those of the WMW probabilistic distributions. It is exactly that last feature that makes our proposal to be easily comprehended and hence effortlessly absorbed. Withal, adapting our reasoning to a proven theoretical framework accelerates enormously the acceptability of our technique. A hidden bonus is that WMW statistics are available for all possible ‘unpaired-sample’ combinations [40]–[41]. This fact becomes a very vital point in generalizing our technique to extend its usefulness to apply synergistically to cubic as well as to nongeometric designs [12]. Ultimately, the strategy to deploy known non-parametric analytics in screening is automatically adaptable to all design resolution types.

**Figure 4:**
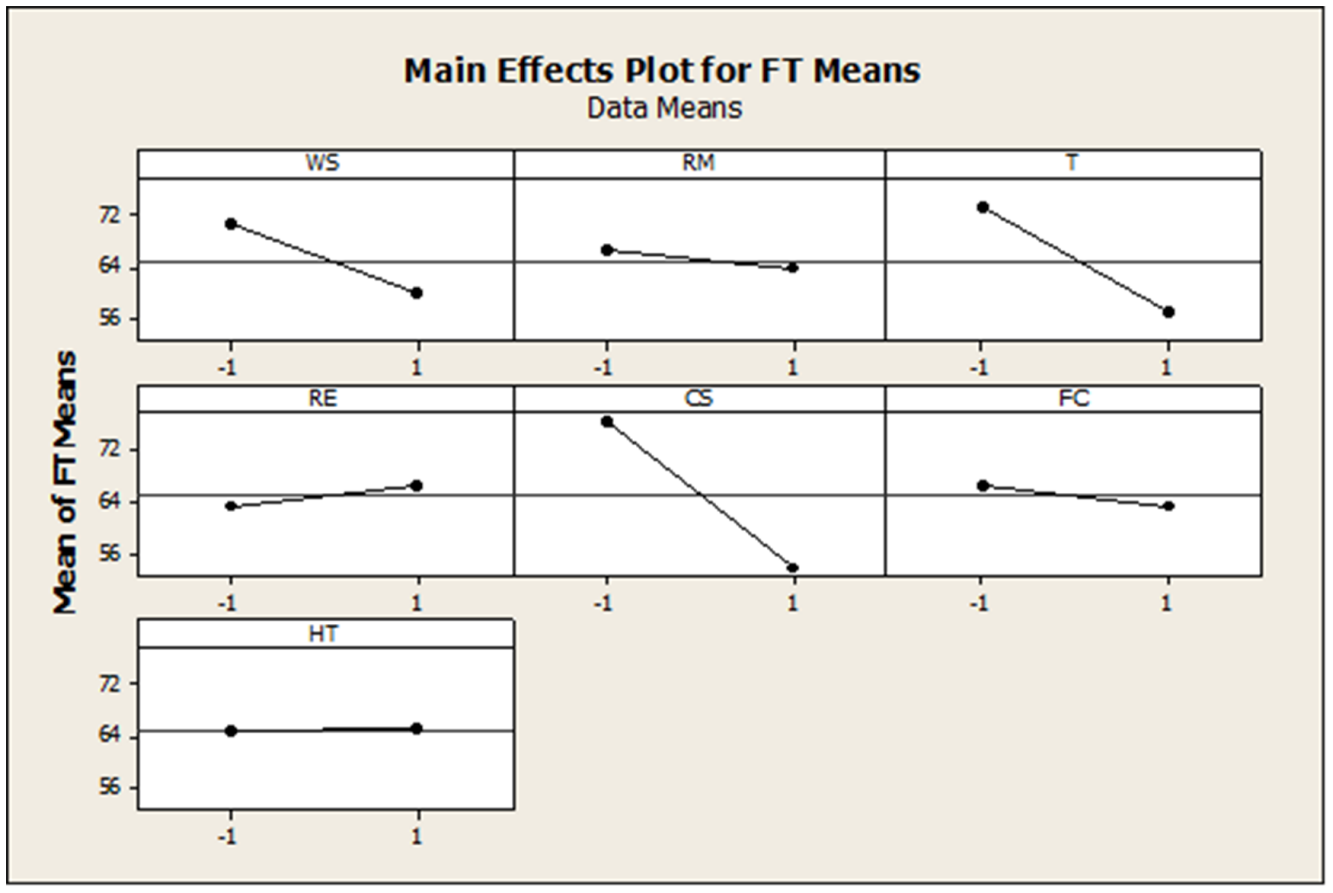
Main effects plot for the means of the filtration time.

To illustrate the potential capabilities of our technique, we tested it against a benchmarked filtration process. We discovered that the three dominant effects in our study's outcomes are congruent to the practical (non-statistical) explanations that have been posted in Box et al. [11]. In Figure 4, we provide a main effects plot that shows actually that WS, T and CS stir up the most variation across their two examined operating endpoints. What the main effects plot does not disclose intelligibly is where to draw the line for discovery in this situation. For example, a plausible philosophical question that may be raised contemplating Figure 4 could be: “Is WS an active factor or a mysterious transition among two possible states?” Another dilemma relates with how truly strong CS is. This can be rephrased in the form of question: “Is CS the preponderant effect?” Despite tracing the steepest slope to CS (Figure 3), the source of this disturbance is statistically indistinguishable with respect to the other two active effects (Table 3). Therefore, information that has been extracted from simple analytics ought to be also stochastically challenged. Additionally, the same group of the three active effects has been confirmed by a highly-specialized method which is based on the powerful maximum-likelihood-ratio principle [46]. However, the maximum-likelihood-ratio method has been formulated to serve only the restricted range of medium-sized unreplicated fractional factorials. Another immediate advantage that needs to be pointed here is that our method converges blindly to the correct final prediction without to be granted a priori refinement for a solution search region as in the Miller approach [46]. Even so, our method clearly outperforms the maximum likelihood ratio test which is operable to an individual error rate of 0.075. Moreover, the best experimentwise error rate for the likelihood ratio test is constrained at a level of 0.25 when in fact over-searching specifically for four active effects. In comparison to our technique (Results section), the Miller method lags in performance with respect to both criteria regarding efficiency in individual and the experimentwise error rates. Definitely, our approach is much simpler and extensible to FFDs larger than the limiting 16-run factorial design that the maximum likelihood ratio technique has been constructed to. By the same token, our technique seems to safeguard more reliably against the possibility of a false discovery event when it is compared with the inferential capabilities of composite non-parametrics. The performance of composite non-parametrics on the same filtration case study has been reported in a past analysis [34]. It was found that composite non-parametrics differentiate on the final prediction with respect to the number of the important effects when considering two different error rates. Depending on the choice of the error rate limit - being either 0.05 or 0.10 - composite non-parametrics underestimated (uncovering two effects) or overestimated (uncovering four effects) the size of the active group. Another gain over the composite non-parametrics method is realized through our proposed method by making explicit the quantification of statistical significance for each influence individually. This means that in an extreme case in which all seven factors were found simultaneously significant to a hypothetical level of 0.05, our method would ostensibly enable mining such overwhelming information. On the contrary, composite non-parametrics would need to lower performance severely to a p-value of 0.333 in order to detect such a rare occurrence (Table 1g in [34]).

Regardless the exclusive reliance on sophisticated tactics for interpreting saturated-unreplicated high-density datasets, it is always instructive to appraise the respective ANOVA results. In Table 5, we list the ANOVA output for the filtration process. At a first glance, we observe how imposing the saturation and unreplication condition on a factorial design is. Only, mean sums of squares (MS) of the studied effects are computable. The synchronous combination of the two conditions intercepts the triggering of F-test comparisons. F-test ratios comprise the primary statistical engine of ANOVA. In lack of any residual degrees of freedom, no estimation for the ‘unknown-and-unknowable’ variation could be concocted. Thus, at this stage, the ANOVA processor downgrades to merely another version of the subjective main effects plot we mentioned above.

**Table 5:** Analysis of Variance for means for the filtration improvement case.

Source	DF^1^	SS^2^	MS^3^	F-test	p-value
WS	1	236.53	236.53	*	*
RM	1	15.4	15.4	*	*
T	1	549.46	549.46	*	*
RE	1	20.16	20.16	*	*
CS	1	1041.96	1041.96	*	*
FC	1	23.46	23.46	*	*
HT	1	0.55	0.55	*	*
Residual Error	0	*	*		
Total	7	1887.53	1887.53		

1 DF = Degrees of Freedom.

2 SS = Sums of Squares.

3 MS = Mean of Sums of Squares.

*Incalculable.

The rank-sum approach adopted in our developments is known to be associated with robust contrasting since it represents location inference based on medians [40]. The median delivers central tendency estimation at the maximum possible breakdown-point performance of 50% which is an essential ingredient in probing new phenomena susceptible to unknown and unknowable intrusions. This prophylactic trait of maximum high breakdown-point performance instilled naturally in the median estimator may not be compensated, under adverse conditions, by a possible advantage in test performance by normal estimators.

Equally important is to realize that the way our technique has been formulated intimately mimics the ANOVA test with respect to the tactic of slicing and contrasting each effect separately. Hence, the test power is uncompromised for every round of the procedure of comparing and eliminating while sizing each influence separately. Nevertheless, we reconfigured the analysis strategy such that to place the contrast focus between the two levels for any given controlling factor. Instead of weighing each effect against the total residual error (as in ANOVA), we disentangled the residual error from any further consideration. By forming the residual vector, *r*′, we allowed screening of the unknown dispersion after the location effects have been figured out [48]. We judged that this was a necessary action for preventing the asymmetric and circumstantial embedding of uncertainty. Surely, our technique is fortified against outliers of unknown origin. This is primarily because of the robust machinery deployed through the fostering of non-parametrics which evidently are deeply rooted at the core of our methodology. Moreover, our introduced residual vector is outfitted with remnant information which is translated to an additional layer of protection for our overall technique. The residual vector furnishes inference against any intruding uncertainty which might stealthily slide into some trial(s) and pollute the dataset. Such behavior is only manifested as an outlier or extreme response in a cautiously contrived data reduction step. To diagnose this occurrence, the stochastic sizing of error is achieved by checking the level of variability of the location estimations across the two factor settings for each factor individually. Furthermore, the basis for sizing up an effect is more comprehensive through our method because of encompassing simultaneously information about the slope of the effect in concert with its relative investigated range. Alternative regression-based methods such as those utilizing the GLM approach are fed only with a slope estimation which endangers the interpretation of subsequent comparisons.

**Figure 5:**
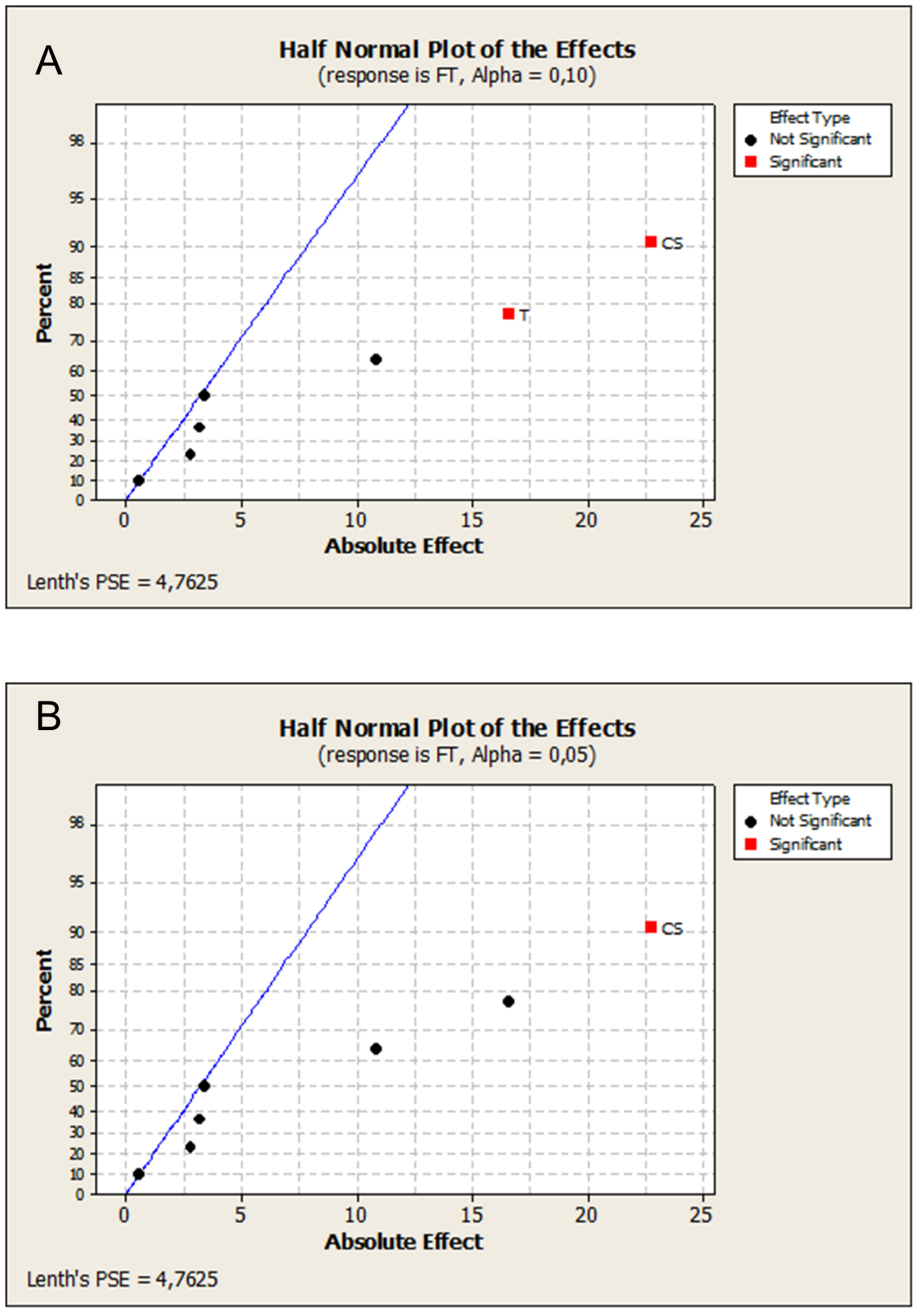
Half-normal plot (MINITAB 16.0) of the effects for the screening of filtration time for significance levels, α = 0.1 (A) and α = 0.05 (B).

**Figure 6:**
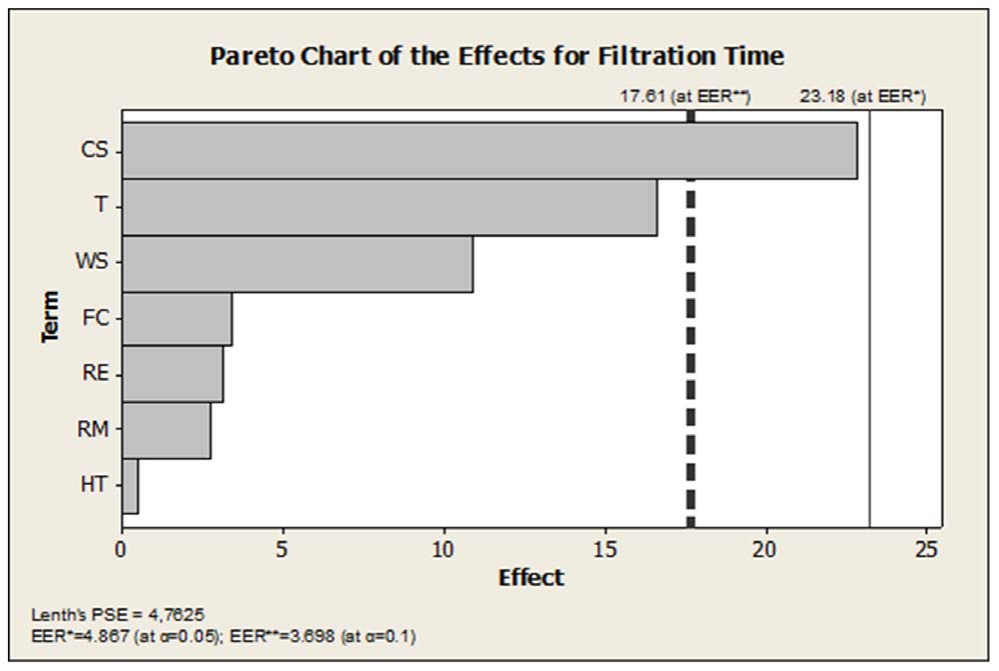
Pareto chart of the effects with assorted EER critical limits according to corrected Lenth method due to Ye and Hamada [49].

Our proposed technique disposes of one of the most controversial assumptions in high-density low-sampling data screening, the so called sparsity assumption [21], [28], [30]. The sparsity assumption causes several impediments in explaining unreplicated-saturated fractional factorial data. First of all, it precludes from the start the possibility that all studied effects might end up to be prevalent. In translation, this a priori condition suggests that some information should always be sacrificed in order to produce any information at all. Therefore, the quality status of a data conversion effort might be assessed only by weighing in sparsity. The crucial predicament which has long been unresolved is how much sparsity should be present in the design to generate acceptable results. This last source of unwavering uncertainty harbors in several sparsity-intensive techniques which are accessible through mainstream data analytics software packages. Astonishingly, no stochastic indicators of sparsity have ever been reported. A favorite commercial combination is the half-normal plot [28] and the Lenth method [30]. In Figure 5, we provide the output (MINITAB 16.0) of the filtration rate screening which automatically returns a result by synthesizing information from the normal plot and the Lenth method. The low detectability at the typical error rate of 0.05 is pronounced in the profiling shown in Figure 5B. Setting the screen at the more crude error rate of 0.1 (Figure 5A), the three active effects have not been recovered entirely. It is striking that contrary to other permutation-based techniques, the rank-sum approach as developed herein is sparsity-free. Hence, it eliminates any dependence on empirical calibration [37]. Calibration is known to relay conflicting conclusions because it considers different cut-off scales devised separately for the individual error rate and the experiment-wise error rate [49]. Depending on how the knowledge worker intends to filter a group of contrasts, effecting a two-way calibration approach may not eventually be a harmonious act. Non-parametric modifications to refine the critical values for the Lenth test - at two principal error rates - have not improved substantially the quality of the prediction [37], [49]. Nevertheless, a better performance standing is attained by resorting to the corrections published by Ye and Hamada [49]. For example, setting the experimentwise error rate at 0.05, the Lenth-test critical value improves to 23.18. However, as it is portrayed in the Pareto chart for the effects in Figure 6, in spite of such refinement, there is no chance for discovering an influence. At an experimentwise error rate which is set at 0.1, the cutoff point now reduces further to a value of 17.6. Even at such a rudimentary selective mode, we observe that two-thirds of the dominant factors have still not been harvested. Otherwise, the EER needs to be raised to a value of 0.30 to enable distinguishing all three active effects. The corresponding IER value predicted by the Ye and Hamada [49] method agrees with our result only when the level is raised for their comparison to at least a value of 0.06. As a side note, the residualization of the original FT-response has been reconfigured in our technique such that exchangeability of the residuals has been maintained for all possible factorial comparisons [38].

We addressed the unreplicated multi-factorial problem which theoretically escapes the crucial reproducibility check. Under such circumstances, there is no guarantee about detecting all unknowable phenomena cryptically permeating the trial runs and distorting asymmetrically the response dataset across the various settings. Nonetheless, our method has been reinforced with a second layer of multi-factorial contrasting which permits the mechanism to profile the residuals for consistency. Flagging the presence of asymmetry within each effect becomes now a systematic process. Certainly, interferences could potentially influence erroneously the rank ordering process by inflating the rank values while incurring redistribution in their ordering status. It might be claimed that the composite nonparametric method [34] is in essence a likewise distribution-free data converter as the one developed in this article. However, the composite nonparametric method lacks of a built-in warning mechanism to fend-off against sneaking uncertainty which spontaneously compounds in asymmetric fashion upon individual data entries. Such situational disturbances might surface from two separate pathways. In one scenario, they might appear to be of unknowable origin due to the lack of awareness of the occurring phenomena by the experimenter.

Unknowable phenomena might not have been explored before at all. The second scenario might be owing to other latent (unknown) variables being present at the moment of conducting trials but for some reason there is no arrangement or no appointed priority for measuring them. Latent variables have a tendency to go undetected in most data processors since there is no provision for inspecting indirectly reproducibility in unreplicated runs. Of course, a superposition of the two stated scenarios may also transpire to a ‘single’ event making matters even more intricate to analyze with standard methods. Hence, our method is equipped to screen through for information against a challenging real-life landscape.

Future work would involve developing new methods that would treat the replicated FFD case by reducing the problem first to a single replicate via a data compressor [50]. Likewise, the multi-response FFD setup would also be confronted by collapsing multiple responses - before analyzing them - to a single master response [51]. In such case, the efficient data processor which we presented in this paper may prove to be handy.

Overall, our technique improves drastically on the recent research for treating saturated-unreplicated datasets with non-parametrics [34]. This is partly attributed to the direct diagnostics now available. By scoping the residual contributions, we verify the potency of the examined effects. It is surprising that by reformulating the general multi-factorial comparison problem such that to be downgraded and attuned to the single unpaired WMW test, the accounting for any degrees of freedom is rendered a meaningless affair. The importance of our method should become more appreciable for highly dense arrays (*k/n*>0.9375). Larger arrays are supplemented with more data per setting thus exploiting the fact that the original WMW distribution scales are known to be even more refined for larger sample sizes.

We reiterate that our procedure is benefited by retaining a transparency code through all phases of data engineering and analysis. This is witnessed from the fact that no complicated routines or novel software are really promoted through this work in order to accomplish the appropriate data conversion steps. A simple spreadsheet application may suffice at most, if it is desired to automate the process for speeding-up recurring estimations. On the other hand, the appropriate tables for the reference distributions of the WMW statistics are available in any introductory statistics textbook or accessible easily from a score of pertinent internet websites.

## Conclusions

In this work, we proposed a method for creating and converting highly-dense, small-sample datasets to concrete information. The method is appropriately constructed for screening the behavior of a process or product characteristic. We showed how a compact dataset planned through a fractional factorial scheme may be decomposed to its constituent effects and then contrasted with the rank sum method of Wilcoxon-Mann-Whitney. The technique proves to be suitable for promoting economic and convenient data processing while providing a combination of highly sought features in modern data mining approaches, i.e. agile analytics and lean reporting. Our technique possesses inherently agile characteristics because the driving engine for our data processor is distribution-free. This means that our profiler will deliver robust information in lack of any prior deep knowledge about the investigated data pattern. Agility has been embedded in our approach to overcome the dataflow deadlock other mainstream multi-factorial analyzers seem to experience under similar conditions. The conversion mismatch due to a cryptic residual error always looms when saturated-unreplicated multi-factorial data collectors are treated by ANOVA or GLM solvers. This syndrome has been prevented through our method. Furthermore, agility is clearly forged in our strategy by removing the binding sparsity assumption. We elucidated the great capabilities for exploiting information in the available dataset under extreme profiling situations. An enormous data volume reduction was initially engineered by sampling though fractional factorials. Information was further squeezed out from an already miniscule dataset by enforcing saturated screening with no provision of replication in the scheduled recipes. We reconfigured the unexplainable error to be gleaned between settings for each effect individually. Thus, we quantified the joint contribution for unknown and unknowable intrusions for each controlling factor. As long as the contrasted error was found to be not significant, only then we advanced in assigning a stochastic interpretation to the status of a particular effect. Lean reporting is assisted by our method because no special software or lengthy algorithms are implicated in the data manipulation and presentation phases. Simplicity and transparency is maintained in all data conversion steps. There is no reason to display the data in many different forms in order to (posteriori) confirm any preset assumptions. Actually, all important information was demonstrated to be relayed through a convenient tabulation.

The technique was applied in a previously examined case study regarding a filtration system for wastewater treatment. The filtration process exemplifies innately a complex phenomenon where fractal and stochastic aspects are thought to be concurrently present. For such a demanding test case, we demonstrated that our approach outperformed several published methods which are considered among the most popular techniques available as well as other more recent technological advances on screening. We compared our method with the half-normal plot, the Lenth method, the ANOVA, the main effects plot, along with the more recent ones such as the Ye-Hamada corrective method, the maximum-likelihood-ratio method and the non-parametric composite effects method.

Summarizing, the proposed approach is a robust and efficient data-mining tool suitable for intricate high-density profiling opportunities. This work attests to that discovery may be assessed eventually with limited data which have been structured in a highly compressed manner. In such circumstances our method is a valuable asset. Undoubtedly, our developments optimize the information value chain in several aspects by: 1) eliminating the need for testing multifarious parametric models, 2) eliminating the need for calibrating error rates, 3) offering great statistical performance for real world paradigms, 4) offering robust screening and decision making by operating distribution-free, 5) promoting efficient, low-cost data engineering and analysis, 6) promoting new-age lean and agile analytics in knowledge discovery.

## Supporting Information

Calculations S1 **Indicative steps to facilitate the understanding of the solution development in the illustrated article paradigm on profiling the filtration process.**
(DOCX)

Table S1 **Filtration experiment inputs** [**11**] **.**
(DOCX)

## References

[1] Dejaegher D, Van der Heyden Y (2011) Experimental designs and their recent advances in set-up, data interpretation, and analytical applications. J Pharmaceut Biomed 56: 141–158. 10.1016/j.jpba.2011.04.023 21632194

[2] De Oliveira FA, de Paiva AP, Lima JWM, Balestrassi PP, Mendes RRA (2011) Portfolio optimization using mixture Design of Experiments: Scheduling trades within electricity markets. Energ Econ 33: 24–32.

[3] Ilzarbe L, Alvarez MJ, Viles E, Tanco M (2008) Practical applications of design of experiments in the field of engineering: A bibliographical review. Qual Reliab Eng Int 24: 417–428.

[4] Katok E (2012) Using laboratory experiments to build better operation management models. Foundations and Trends® in Technology, Information and Operations Management 5: 1–88.

[5] Knight R, Jansson J, Field D, Fierer N, Desai N, et al (2012) Unlocking the potential of metagenomics through experimental design. Nat Biotechnol 30: 513–520. 22678395 10.1038/nbt.2235 PMC4902277

[6] Kuhfeld WF, Tobias RD (2005) Large factorial designs for product engineering and marketing research applications. Technometrics 47: 132–141.

[7] List JA, Sadoff S, Wagner M (2011) So you want to run an experiment, now what? Some simple rules of thumb for optimal experimental design. Exp Econ 14: 439–457.

[8] Oberg AL, Vitek O (2009) Statistical design of quantitative mass spectrometry-based proteomic experiments. J Proteome Res 8: 2144–2156. 19222236 10.1021/pr8010099

[9] Singh B, Kumar R, Ahuja N (2005) Optimizing drug delivery systems using systemic “design of experiments”: Part I: Fundamental aspects. Crit Rev Ther Drug 22: 27–105. 10.1615/critrevtherdrugcarriersyst.v22.i1.20 15715503

[10] Fisher RA (1990) Statistical methods, Experimental Design and Scientific inference. New York: Oxford University Press.

[11] Box GEP, Hunter JS, Hunter WG (2005) Statistics for Experimenters: Design, Innovation, and Discovery. Hoboken, NJ: Wiley.

[12] Montgomery DC (2009) Design and Analysis of Experiments. Hoboken, NJ: Wiley.

[13] Thomke SH (1998) Managing experimentation in the design of new products. Manage Sci 44: 743–762.

[14] Nair V, Strecher V, Fagerlin A, Ubel P, Resnicow K, et al (2008) Screening experiments and the use of fractional factorial designs in behavioral intervention research. Am J Public Health 98: 1354–1359. 18556602 10.2105/AJPH.2007.127563 PMC2446451

[15] Rufer A, Reschetilowski W (2012) Application of design of experiments in heterogeneous catalysis: Using the isomerization of n-decane for a parameter screening. Chem Eng Sci 75: 364–375.

[16] Swalley SE, Fulghum JR, Chambers SP (2006) Screening factors effecting a response in soluble protein expression: Formalized approach using design of experiments. Anal Biochem 351: 122–127. 16434014 10.1016/j.ab.2005.11.046

[17] Zhang XD, Yang XC, Chung N, Gates A, Stec E, et al (2006) Robust statistical methods for hit selection RNA interference high-throughput screening experiments. Pharmacogenomics 7: 299–309. 16610941 10.2217/14622416.7.3.299

[18] Hoaglin DC, Mosteller F, Tukey JW (2000) Understanding Robust and Exploratory Data Analysis. Hoboken, NJ: Wiley-Interscience.

[19] Berk KN, Picard RR (1991) Significance tests for saturated orthogonal arrays. J Qual Technol 23: 79–89.

[20] Haaland PD, O'Connell MR (1995) Inference for effect-saturated fractional factorials. Technometrics 37: 82–93.

[21] Kunert J (1997) On the use of the factor-sparsity assumption to get an estimate of the variance in saturated designs. Technometrics 39: 81–90.

[22] Voss DT (1999) Analysis of orthogonal saturated designs. J Stat Plan Infer 78: 111–130.

[23] Wu SS, Wang W (2007) Step-up simultaneous tests for identifying active effects in orthogonal saturated designs. Ann Stat 35: 449–463.

[24] Al-Shiha AA, Yang S-S (2000) Critical values and some properties of a new test statistic for analyzing unreplicated factorial experiments. Biometrical J 42: 605–616.

[25] Birnbaum A (1959) On the analysis of factorial experiments without replication. Technometrics 1: 343–357.

[26] Box GEP, Meyer RD (1986) An analysis for unreplicated fractional factorials. Technometrics 28: 11–18.

[27] Brenneman WA, Nair VN (2001) Methods for identifying dispersion effects in unreplicated factorial experiments: A critical analysis and proposed strategies. Technometrics 43: 388–405.

[28] Daniel C (1959) Use of half-normal plots in interpreting factorial two-level experiments. Technometrics 1: 311–341.

[29] Holms AG, Berrettoni JN (1969) Chain-pooling ANOVA for two-level factorial replication-free experiments. Technometrics 11: 725–746.

[30] Lenth RV (1989) Quick and easy analysis of unreplicated factorials. Technometrics 31: 469–473.

[31] Chen Y, Kunert J (2004) A new quantitative method for analyzing unreplicated factorial designs. Biometrical J 46: 125–140.

[32] Hamada M, Balakrishnan N (1998) Analyzing unreplicated factorial experiments: A review with some new proposals. Stat Sinica 8: 1–41.

[33] Besseris GJ (2008) Analysis of an unreplicated fractional-factorial design using nonparametric tests. Quality Engineering 20: 96–112.

[34] Besseris GJ (2009) Order statistics for two-level, eight-run saturated-unreplicated fractional-factorial screening. Quality Engineering 21: 416–431.

[35] Aguirre-Torres V, de la Vara R (2012) A robust analysis of unreplicated factorials. Appl Stoch Model Bus 28: 194–205.

[36] Costa N, Pereira ZL (2007) Decision-making in the analysis of unreplicated factorial designs. Quality Engineering 19: 215–225.

[37] Loughin TM, Noble W (1997) A permutation test for effects in an unreplicated factorial design. Technometrics 39: 180–190.

[38] Pesarin F, Salmaso L (2002) Exact permutation tests for unreplicated factorials. Appl Stoch Model Bus 18: 287–299.

[39] Farias VF, Jagabathula S, Shah D (2013) A Nonparametric Approach to Modeling Choice with Limited Data Manage Sci doi: 10.1287/mnsc.1120.1610.

[40] Wilcoxon F (1945) Individual comparisons by ranking methods. Biometrics 1: 80–83.

[41] Mann HB, Whitney DR (1947) On a test of whether one of two random variables is stochastically larger than the other. Ann Math Stat 18: 50–60.

[42] Hemelrijk J (1952) Note on Wilcoxon's two-sample test when ties are present. Ann Math Stat 23: 133–135.

[43] Hamilton RA (1984) What will we do when the well runs dry? Harvard Bus Rev 62: 28–30.

[44] Kurland NB, Zell D (2010) Water and business: A taxonomy and review of the research. Organ Environ 23: 316–353.

[45] Pignon F, Magnin A, Piau J-M, Cabane B, Aimar P, et al (2000) Structural characterization of deposits formed during frontal filtration. J Membrane Sci 174: 189–204.

[46] Miller A (2005) The analysis of unreplicated factorial experiments using all possible comparisons. Technometrics 47: 51–63.

[47] Benjamini Y, Hochberg Y (1995) Controlling the false discovery rate: a practical and powerful approach to multiple testing. J R Statist Soc B 57: 289–300.

[48] Schoen ED (2004) Dispersion-effects detection after screening for location effects in unreplicated two-level experiments. J Stat Plan Infer 126: 289–304.

[49] Ye KQ, Hamada M (2000) Critical values of the Lenth method for unreplicated factorial designs. J Qual Technol 32: 57–66.

[50] Besseris GJ (2012) Profiling effects in industrial data mining by non-parametric DOE methods: An application on screening checkweighing systems in packaging operations. Eur J Oper Res 220: 147–161.

[51] Besseris GJ (2012) Multi-response multi-factorial master ranking in non-linear replicated-saturated DOE for qualimetrics. Chemometr Intell Lab 116: 47–56.

