# Supplementary material for: A Distribution-Free Multi-Factorial Profiler for Harvesting Information from High-Density Screenings
Source: PLoS One. 2013 Aug 29;8(8):e73275. doi: 10.1371/journal.pone.0073275 (PMC3756950; doi:10.1371/journal.pone.0073275)
Supplement: Calculations S1 — Indicative steps to facilitate the understanding of the solution development in the illustrated article paradigm on profiling the filtration process. (DOCX) [file pone.0073275.s001.docx]

1. The grand median for the available data from Table 2 is *M*=Median {68.4, 77.7, 66.4, 81.0, 78.6, 41.2, 68.7, 38.7} = 68.55.
2. The median settings are computed from the participating entries. For instance, to form the median $M_{l}^{+}$ for WS we pick the values that contribute to the same level, i.e. median {77.7, 81.0, 41.2, 38.7}=59.45. Similarly, to get the median settings for$M_{l}^{-}$, we compute median{68.4, 66.4, 78.6, 68.7}=68.55. The last two values are listed in the respective columns in Table 3 next to the corresponding label, WS. This way the rest of the six rows are completed.
3. To form the quantities $D_{l}^{+}$and$D_{l}^{-}$, we simply subtract the grand median from the respective $M_{l}^{+}$and$M_{l}^{-}$ values, i.e. for WS this computes to be: 59.5-68.55=-9.05 and 68.55-68.55=0, respectively (Table 3).
4. To form the pseudo-response *y’* for WS for the first run (Table 4) we need to compute the error $\varepsilon_{i_{1},i_{2},...,i_{k}}$:
   1. For Run #1 in Table 2 the $\varepsilon_{i_{1},i_{2},...,i_{k}}$is computed as follows: $\varepsilon_{i_{1},i_{2},...,i_{k}}$= FT_1_-*M*-$D_{WS}^{-}$-$D_{RM}^{-}$-$D_{T}^{-}$-$D_{RE}^{+}$-$D_{CS}^{+}$-$D_{FC}^{+}$-$D_{HT}^{-}$ = 68.4-68.55-0-4.5-4.5-4.95-(-14.75)-0-0=0.65
   2. For Run #1 for WS the *y’* = *M* +$D_{WS}^{-}$+ $\varepsilon_{i_{1},i_{2},...,i_{k}}$= 68.55-0+0.65 = 69.2 (Table 4).
5. We may now form the residual-response *y”* for the first run (Table 4):

For Run #1, the $y''=M+\varepsilon_{i_{1},i_{2},...i_{l}...,i_{k}}\text{ }$*=* 68.55+0.65=69.2(Table 4).

1. Now rank ordering is easily effected on the columns in the left hand side. To rank the data for WS we simply start counting from the entry with the smallest magnitude. For example, from Table 4, for the WS pseudo-vector *y’* is written in ranked form *r* as follows initiating the counting from the entry 56.6:

$$\left[ \begin{matrix} 69.2 \\ \begin{matrix} 56.6 \\ \begin{matrix} 71.2 \\ \begin{matrix} 59 \\ \begin{matrix} 65.7 \\ \begin{matrix} 62.1 \\ \begin{matrix} 74.7 \\ 59.6 \end{matrix} \end{matrix} \end{matrix} \end{matrix} \end{matrix} \end{matrix} \end{matrix} \right]\underset{\to}{rank-ordering}\left[ \begin{matrix} 6 \\ \begin{matrix} 1 \\ \begin{matrix} 7 \\ \begin{matrix} 2 \\ \begin{matrix} 5 \\ \begin{matrix} 4 \\ \begin{matrix} 8 \\ 3 \end{matrix} \end{matrix} \end{matrix} \end{matrix} \end{matrix} \end{matrix} \end{matrix} \right]$$

2. To form the rank sums simply retain the smaller of the two rank sums for a given effect. For example, for WS we see that to form T_WS+_ we need to consider runs #2, 4, 6 and 8 (Table 2). These runs correspond to ranks 1,2,4,3 in Table 4, in the ranked response r-column allocated for WS. Their sum is thus T_WS+_=1+2+4+3=10 (Table 3).
3. According to reference #41 in the manuscript for equal sample sizes of 4 the two-sided p-value for a rank-sum of 10 is 0.029 (=*p_T_*) in Table 3.
4. Similarly, we obtain p-values for the rest of the effects and the residual-response.
