# Supplementary material for: A Distribution-Free Multi-Factorial Profiler for Harvesting Information from High-Density Screenings
Source: PLoS One. 2013 Aug 29;8(8):e73275. doi: 10.1371/journal.pone.0073275 (PMC3756950; doi:10.1371/journal.pone.0073275)
Supplement: Table S1 — Filtration experiment inputs [11] . (DOCX) [file pone.0073275.s002.docx]

| *Controlling factor* |  | *Setting Selection* | |
| --- | --- | --- | --- |
|  |  | - | + |
| Water Supply |  | town reservoir | well |
| Raw Material |  | on site | other |
| Temperature |  | low | high |
| Recycle |  | yes | no |
| Caustic Soda |  | fast | slow |
| Filter Cloth |  | new | old |
| Holdup Time |  | low | high |
